# *In vitro* evaluations on canine monocyte-derived dendritic cells of a nanoparticles delivery system for vaccine antigen against *Echinococcus granulosus*

**DOI:** 10.1371/journal.pone.0229121

**Published:** 2020-02-26

**Authors:** Nadège Milhau, Eyad Almouazen, Sylvie Bouteille, Imène Hellel-Bourtal, Samira Azzouz-Maache, Uruguaysito Benavides, Anne-Françoise Petavy, Thierry Marchal

**Affiliations:** 1 Université de Lyon, VetAgro Sup, UPSP ICE 2011.03.101, Marcy L’Etoile, France; 2 Univ Lyon, Université Claude Bernard Lyon 1, CNRS, LAGEPP UMR 5007, Villeurbanne, France; 3 ISPB-Faculté de Pharmacie, Université Claude-Bernard Lyon 1, Lyon, France; 4 Université de Lyon, VetAgro Sup, Laboratoire d’Histopathologie, Marcy L’Etoile, France; 5 Institut de recherche pour le développement (IRD), UMR InterTryp IRD/CIRAD, campus international de Baillarguet, Montpellier, France; 6 Immunology Department, Faculty of Veterinary, Universidad de la República, Montevideo, Uruguay; INSERM, FRANCE

## Abstract

Since dogs play a central role in the contamination of humans and livestock with *Echinococcus granulosus*, the development of an effective vaccine for dogs is essential to control the disease caused by this parasite. For this purpose, a formulation based on biodegradable polymeric nanoparticles (NPs) as delivery system of recombinant *Echinococcus granulosus* antigen (tropomyosin *Eg*Trp) adjuved with monophosphoryl lipid A (MPLA) has been developed. The obtained nanoparticles had a size of approximately 200 nm in diameter into which the antigen was correctly preserved and encapsulated. The efficiency of this system to deliver the antigen was evaluated *in vitro* on canine monocyte-derived dendritic cells (cMoDCs) generated from peripheral blood monocytes. After 48 h of contact between the formulations and cMoDCs, we observed no toxic effect on the cells but a strong internalization of the NPs, probably through different pathways depending on the presence or not of MPLA. An evaluation of cMoDCs activation by flow cytometry showed a stronger expression of CD80, CD86, CD40 and MHCII by cells treated with any of the tested formulations or with LPS (positive control) in comparison to cells treated with PBS (negative control). A higher activation was observed for cells challenged with *Eg*Trp-NPs-MPLA compared to *Eg*Trp alone. Formulations with MPLA, even at low ratio of MPLA, give better results than formulations without MPLA, proving the importance of the adjuvant in the nanoparticles structure. Moreover, autologous T CD4+ cell proliferation observed in presence of cMoDCs challenged with *Eg*Trp-NPs-MPLA was higher than those observed after challenged with *Eg*Trp alone (p<0.05). These first results suggest that our formulation could be used as an antigen delivery system to targeting canine dendritic cells in the course of *Echinococcus granulosus* vaccine development.

## Introduction

Echinococcal hydatidosis is a parasitic disease caused by the larvae of the dog tapeworm *Echinococcus granulosus*. This parasite requires two successive hosts for its complete life cycle [1]. The definitive hosts) are domestic carnivores, such as dogs, in which adult tapeworms inhabit the small intestine and deliver eggs excreted with the feces. The intermediate hosts are herbivorous animals, such as sheep, goats, bovines or pigs. Humans are accidental intermediate hosts, infected by contaminated food or water with the parasite eggs. In human and herbivorous disease, larvae localized in the liver (75% of cases) or lungs (5–15% of cases) develop in cysts, which can lead to allergic reactions or even death [1,2]. *Echinococcus granulosus* is present on all continents. In endemic areas (South America, Asia, and North and East Africa), this zoonotic disease represents a serious public health problem with more than 50 cases per 100 000 persons per years. In farm animals, cystic Echinococcosis results in herd and economic losses [2,3]. Dog’s vaccination against the parasite appears to be an effective means of decreasing this disease [3].

For this purpose, several antigens have been previously evaluated as potential immunogen for a vaccine. For livestock, the *Eg*95 protein shows high protection in both sheep [4] and bovines [5]. However, polymorphism in the *Eg*95 sequences was found in different *E*. *granulosus* isolates; which limits vaccine efficiency [6]. For dogs, Zhang et al. showed that EgM123 and EgM9 recombinant proteins emulsified with Freud’s adjuvant induced a high level of protection in terms of both suppression of worm growth and egg development [7]. The authors confirmed this result recently with the same recombinant proteins and Quil A as adjuvant [8]. In our group, we were interested in two antigens, *Eg*Trp (*E*. *granulosus* tropomyosin) and *Eg*A31 (*E*. *granulosus* paramyosin). The immunogenicity of both antigens has been previously demonstrated in mice by the production of high specific antibody titers of IgG1 and IgA and IL-12, IFN, IL-10 and IL-6 cytokines in immunized animals [9]. In a previous work, we used these two recombinant proteins expressed in a Salmonella vaccine vector for a dog vaccine formulation, which was tested in two parallel experimental trials performed in Morocco and Tunisia. The results showed a decrease of more than 70% in the cestode burden in dogs and a slower development rate for parasites in vaccinated animals [10]. However, despite the high level of protection induced, the use of bacteria as a delivery system could be considered as an environmental and ethical problem. For these reasons, developing a vaccine with an alternative delivery system, such as nanoparticles (NPs), could be a relevant strategy to induce protective immune responses in dogs.

Polymeric nanoparticles as antigen delivery systems have been tested in vaccination against different diseases, such as Listeriosis [11], Malaria [12] and Dengue disease [13], with hopeful results. The two most commonly biodegradable polymers used in vaccine applications are polylactic-co-glycolic acid (PLGA) and polylactic acid (PLA). As these polymers have been approved by the US Food and Drug Administration (FDA) for human use, they have been intensively explored in human and mouse vaccine models [14]. These particles, with sizes ranging between 20 and 200 nm, offer many advantages, such as improved stability, enhanced immunogenicity and sustained release profile, thus limiting the number of administrations in vaccination protocols. Furthermore, they can be engineered to enhance the targeting of antigen presenting cells, such as Dendritic Cells (DCs), and therefore initiate a strong and specific immune response [15,16]. Indeed, DCs control a substantial part of the adaptive immune response by internalizing antigens *via* different receptors and processing them through the MHC pathways [17]. To improve the efficiency of nanoparticle vaccines, numerous authors use ligands that are able to specifically recognize some of these receptors, such as monophosphoryl lipid A (MPLA), a derivative of non-toxic LPS targeting TLR4 receptors on DCs. This “danger signal” induces DCs maturation, resulting in the upregulation of the expression of MHC II and the costimulatory molecules CD80, CD86 and CD40 as well as the secretion of inflammatory cytokines [18,19].

In this context, the aim of this study was to evaluate the ability of a nanoparticles delivery system to activate DCs in the course of *Echinococcus granulosus* vaccine development. For this, we used biodegradable PLA nanoparticles as vehicle, MPLA as adjuvant and the recombinant *Eg*Trp protein as antigen. In our final vaccine formulation, the two previously identified antigens by our team (*Eg*Trp and *Eg*A31) will be included. However, the first results showed that these two antigens cannot be encapsulated together into the same nanoparticle. This is why, in order to simplify the experiments, we tested only one delivery system containing the *Eg*Trp antigen in this preliminary study. Physicochemical properties of the nanoparticles obtained as well as the integrity of the encapsulated antigen were first evaluated. PLA-NPs uptake and cytotoxic effect were then studied in cMoDCs cultures. Finally, the cMoDCs maturation and the ability of cMoDCs challenged with PLA-NPs to induce T cell priming were analyzed.

## Materials and methods

### Antigen production and purification

The *Eg*Trp recombinant proteins were produced and purified as previously described [20]. Briefly, *pQIA-Eg*Trp was expressed in the M15 (pREP4) (Qiagen) *Escherichia col*i strain. Bacterial cells containing the relevant plasmid were cultured at 37°C in yeast extract and tryptone medium supplemented with ampicillin/kanamycin, up to an OD600 nm of 0.5 to 0.7. *Eg*Trp expression was induced by the addition of 1.5 mM isopropyl β-D-1-thiogalactopyranoside, and the bacteria were incubated for an additional 3h. The bacteria were then lysed in cell lysis buffer (50 mM phosphate buffer pH 8, 300 mM NaCl, 20 mM imidazole) and ruptured by sonication. Cellular debris were removed by centrifugation at 15 000 x g for 30 minutes at 4°C, and the supernatant was loaded onto a His-Trap 5 mL column (GE Healthcare). Elution was performed with a buffer containing increasing concentrations of imidazol (100 mM, 200 mM, 300 mM, and 500 mM). The fractions were analyzed by SDS-PAGE and MALDI-TOF mass spectrometry.

### Nanoparticle preparation

Nanoparticle formulations were prepared by a double emulsion-evaporation method previously described by Bilati et al. [21], with some modifications. Briefly, 1 g of PLA (Evonik Biomaterials, Germany) was dissolved in 20 mL of ethyl acetate to a final proportion of 5%. Then, quantities of MPLA (from *Salmonella enterica* serotype Minnesota Re595, Sigma-Aldrich, France) ranging between 0.25 mg to 2 mg were solubilized in a methanol-chloroform mixture (1:4 –V/V) and introduced into the organic phase with magnetic stirring. The first emulsion was made by adding the internal aqueous phase (4 mg of antigen *Eg*Trp in 2 mL of its elution buffer) dropwise to the organic phase under magnetic stirring combined with sonication at an amplitude of 25% (S-4000, Misonix) for two minutes. The second emulsion was created by adding the first emulsion dropwise to 40 mL of 2% polyvinyl alcohol aqueous solution (Sigma-Aldrich, France) under magnetic stirring combined with sonication at an amplitude of 25% for another two minutes in an ice bath. The preparation was then magnetically stirred for 20 minutes. Finally, the organic solvent was evaporated at 40°C and 200 mbar using a rotary evaporator (Rotavapor^®^ RE-140, Büchi, Switzerland). The final nanoparticle suspensions were adjusted to 40 ml with purified water to obtain final concentrations of *Eg*Trp antigen at 0.1 mg/ml and MPLA at 6.12 μg/ml, 12.5 μg/ml, 25 μg/ml and 50 μg/ml, corresponding to the 0.025%, 0.05%, 0.1% and 0.2% loading ratios, respectively.

Blank-NPs were prepared in similar conditions by replacing the antigen solution with 2 mL of distilled water. For the cell internalization experiment, iron (II, III) oxide nanoparticles with an average diameter of 5 nm were used as markers. Iron oxide (Sigma-Aldrich, France) was incorporated into the nanoparticles by adding 2 mL of aqueous iron oxide suspension (5 mg/mL) in place of the aqueous internal phase during the first emulsion preparation.

### Nanoparticle characterization and encapsulated antigen integrity evaluation

The particle size, size distribution and zeta potential of the nanoparticles in an aqueous environment were investigated by the dynamic light scattering technique, using a Zetasizer Nano ZS90 (Malvern Instruments, UK). To evaluate the encapsulated protein quantity, the nanoparticle suspension was centrifuged at 28 000 x g for 30 minutes to separate free protein in the supernatant from encapsulated protein in the pellet. The encapsulated proteins were then released from the particles by dissolving the polymer with acetonitrile. The amount of protein in the nanoparticle suspension, in the supernatant, and in the pellet were quantified by Bradford reagent (Sigma-Aldrich). To evaluate the integrity of the encapsulated protein, a 12.5% SDS-PAGE stained with Coomassie Blue was performed. Finally, to evaluate the immunogenicity of the encapsulated proteins, a Western Blot with an anti-*Eg*Trp rabbit immune serum was performed with recombinant *Eg*Trp treated or not with acetonitrile as controls.

In order to estimate the encapsulation efficiency of MPLA, the nanoparticle suspension was centrifuged at 28 000 x g for 30 minutes to separate free MPLA in the supernatant from encapsulated MPLA in the pellet. The pellet was then dissolved with acetonitrile. The MPLA extracts form the supernatant and the pellet were analyzed using high-pressure liquid chromatography. The HPLC set up from Waters (Saint Quentin en Yvelines, France) was composed of a Waters 717 autosampler injector, a Waters 600 pump with gradient controller and an ELSD detector (2424 ELS Detector Waters, USA). The analysis was done using the X-Terra C8 column (3.9 x 150 mm; particle size 5 μm, Waters). The mobile phase consisted of methanol-water (90:10, v/v) with a flow rate of 1.5 mL/min. The ELSD conditions were: drift tube temperature of 50°C, nitrogen gas pressure 50 psi, and gain 200 bar. The injection volume was 30 μL. A standard curve was established between the 0.5 and 50 μg/mL.

### Generation of canine monocyte-derived dendritic cells (cMoDCs)

All the protocols used in this study have been submitted to the VetAgro Sup animal ethics committee (Lyon, France) and have been approved. Blood samples were taken from adult beagles at the Claude Bourgelat Institute of the VetAgro-Sup Veterinary Campus of Lyon. Between 40 and 50 mL of blood were drawn from the jugular vein of each dog into an ACD tube (BD Vacutainer Systems, Plymouth, UK) on day 0. The blood samples were applied to Ficoll density gradients of 1.077 (Eurobio, France) after PBS dilution (1:3 –V/V) and centrifuged at 600 x g for 30 minutes at 25°C. Peripheral blood mononuclear cells (PBMCs) were harvested from the interface, washed three times with PBS, and then suspended in RPMI-1640 medium supplemented with 10% fetal calf serum (FCS) and 2% penicillin/streptomycin (Eurobio, France), referred as complete RPMI medium.

To generate *in vitro* cMoDCs from peripheral blood precursors, we used two different methods already described in the literature: the adherence method [22] and the positive magnetic-sorting selection method [23]. For the adherence method, PBMCs were allowed to adhere to a 6-well tissue culture plate for 24 h at 37°C. Non-adherent cells were then gently removed from the culture supernatant, and the adherent cells were cultured for another five days in complete RPMI medium supplemented with 53 ng/mL canine GM-CSF and 17 ng/mL canine IL-4 (R&D Systems, France). For the positive magnetic-sorting selection method, a positive selection of CD14+ cells from PBMCs was performed with the MACS system (Miltenyi Biotec, France). Freshly prepared PBMCs were incubated for 30 minutes at 4°C with a mouse anti-bovine CD14 (CAM36A, CliniSciences, France), washed with PBS, incubated for 30 minutes at 4°C with the secondary goat anti-mouse microbeads (Miltenyi Biotec, France) and then placed onto an MS column according to the manufacturer’s instructions. After several washing steps, CD14^+^ cells were obtained with purity above 90%, as determined by flow cytometry. The resulting cells were suspended in complete RPMI medium supplemented with 53 ng/mL canine GM-CSF and 17 ng/mL canine IL-4 and cultured in a 12-well plate for six days at 37°C.

For these two protocols, morphological changes from monocytes (day 0) to cMoDCs (day 6) were characterized by flow cytometry analysis (size and granulometry) and microscopic light observation after cytocentrifugation and May Grünewald Giemsa staining (Microm Microtech, France, F/010253). A comparison of cMoDCs obtained with these two protocols is shown in supplemental data (S1 Fig).

### Evaluation of nanoparticle internalization by cMoDCs

To evaluate nanoparticle internalization, cMoDCs were first incubated with iron-labeled nanoparticles containing or lacking MPLA at 0.1% at a final dilution of 1/100 in the cell culture medium. After 2 h, 24 h and 48 h of contact, the cells were cytocentrifuged for 5 minutes at 500 rpm on microscope slides using a Cytospin-2 centrifuge, and Perls staining was performed according to the manufacturer’s instructions to detect iron in the cells (Microm Microtech, France, F/010236). Morphological changes in cMoDCs were observed after May Grünewald Giemsa staining (Microm Microtech, France, F/010253). In a second step, to optimize the MPLA concentration, the same experiment was performed with 24 h of contact using four nanoparticle formulations with MPLA at 0.025%, 0.05%, 0.1% and 0.2%.

### Cytotoxicity of the nanoparticle formulations

To evaluate the cytotoxicity of the formulations, cMoDCs were incubated with nanoparticles containing or lacking MPLA at 0.025%, 0.05%, 0.1% and 0.2% for 48 h. The cells were then harvested from the cultures by trypsin treatment (Eurobio, France), stained with 5 μL of FITC Annexin V and 5 μL of propidium iodide (FITC Annexin V Apoptosis Detection Kit I; BD Biosciences, France) and directly analyzed by flow cytometry to evaluate apoptosis and necrosis.

### Evaluation of cMoDCs activation

To evaluate cMoDCs activation, the expression levels of activation markers were determined by flow cytometry after direct immunostaining of the cMoDCs using anti-CD40 (LOB7/6, Serotec), anti-MHC II (YKIX334.2, Serotec), anti-CD80 (16-10A1, Biolegend), and anti-CD86 (IT2.2, Biolegend) monoclonal antibodies. For this experiment, the cells were stained for 30 minutes at 4°C with the antibody labeled with FITC or PE or the isotype control, washed with PBS and analyzed with an Accuri cytometer (Becton Dickinson). Two parameters were investigated: the percentage of positive cells and mean fluorescence intensity (MFI), which reflects the quantitative expression of the marker on the cell surface. A fold increase in MFI, which reflects cell activation, was calculated as follows: (MFI of challenged cells–MFI of PBS)/MFI of PBS.

To optimize the quantity of MPLA used in the final formulation, we evaluated the expression of the activation markers 24 h after challenging the cMoDCs with nanoparticles containing four different MPLA loading ratios: 0.025%, 0.05%, 0.1% and 0.2%. We then evaluated the expression of the activation markers on cMoDCs 24 h after being challenged with one of the following formulations: blank-NPs, *Eg*Trp alone, *Eg*Trp-NPs, NPs-MPLA and *Eg*Trp-NPs-MPLA, using the best MPLA loading ratio previously selected. This experiment was repeated on cMoDCs on four different dogs.

### Autologous TCD4+ cells proliferation assay

For proliferation assay, cMoDCs were challenged 24h with LPS or with one of the following formulations: blank-NPs, *Eg*Trp alone, *Eg*Trp-NPs, NPs-MPLA and *Eg*Trp-NPs-MPLA, and then washed. Fresh autologous TCD4+ cells were purified from dogs’ PBMCs using magnetic beads (Miltenyi Biotec, France) and a rat anti-dog CD4+ antibody (Biorad, France) as previously described. The selected cells, with a purity over 95%, were suspended in complete RPMI medium and co-cultured 5 days with cMoDCs at a ratio of 10/1 in 96-well flat-bottom plates. TCD4+ cells alone or in the presence of PHA (Sigma-Aldrich, France) at 10μg/ml were used as negative or positive control, respectively. After 5 days of incubation, 10μl CCK8 (Sigma-Aldrich, France) were added into each well and incubated at 37°C for 2h. The absorbance was measured at 450 nm using a microplate reader (MultiSkan, Thermofisher). Results were expressed as mean ± standard deviation. Experiments were performed in triplicate on cells obtained from three different dogs.

### Statistical analysis

Differences between formulations in all tests were analyzed with a paired Student’s t test. Graphs and statistical analyses were performed using GraphPad Prism version 7 software. P-values ≤ 0.05 were considered significant.

## Results

### Nanoparticle formulation and characterization

**Fig 1:**
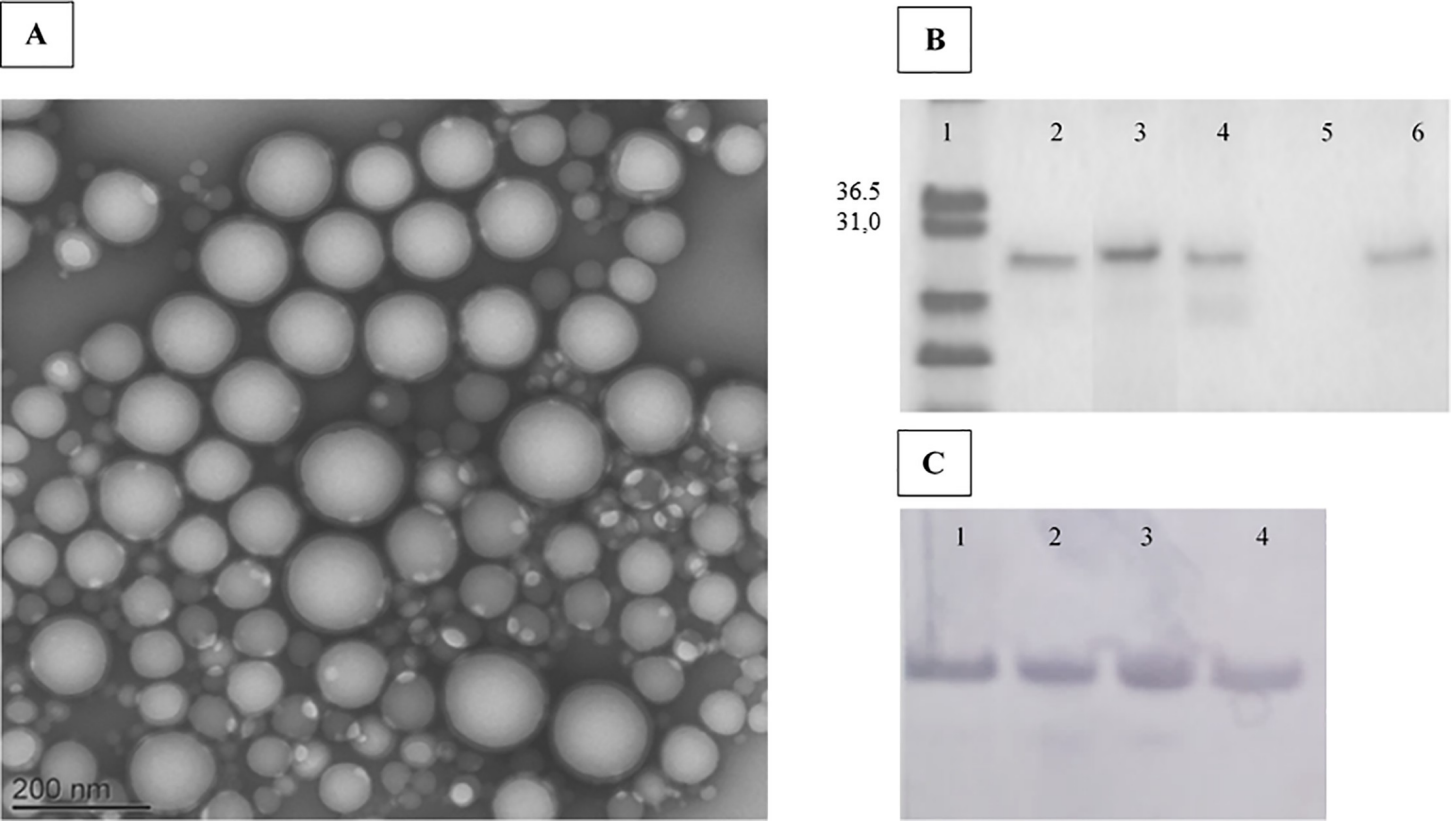
Nanoparticles characterization. (A) Transmission electronic microscopy image of the *Eg*Trp-NPs-MPLA formulation. (B) SDS page analysis after Coomassie Blue staining of *Eg*Trp extracted from NPs. line 1: molecular weight marker), line 2: *Eg*Trp (positive control), line 3: *Eg*Trp treated with acetonitrile (control of extraction step), line 4: total *Eg*Trp-NPs-MPLA suspension after treatment with acetonitrile, line 5: supernatant, line 6: pellet of NPs after treatment with acetonitrile. (C) Western blot analysis of *Eg*Trp extracted from NPs and revealed with an anti- *Eg*Trp rabbit serum. line 1: *Eg*Trp treated with acetonitrile (control), lines 2, 3 and 4: *Eg*Trp obtained after treatment with acetonitrile of 3 different preparations of *Eg*Trp-NPs-MPLA.

The double emulsification-evaporation technique performed to encapsulate the antigen showed a good encapsulation efficiency of approximately 80% for both the *Eg*Trp-NPs and *Eg*Trp-NPs-MPLA. The nanoparticles were negatively charged and had a monodispersed size with a mean size of approximately 200 nm without differences between the blank-NPs and *Eg*Trp-NPs. Indeed, the size ranged between 184 nm and 219 nm for the Blank-NPs, between 165 nm and 230 nm for the *Eg*Trp-NPs, and between 179 nm and 190 nm for the *Eg*Trp-NPs-MPLA (Table 1). The results with the *Eg*Trp-NPs-MPLA showed that adding the adjuvant slightly reduced the nanoparticle size and enhanced antigen encapsulation efficiency (Table 1). Transmission electron microscope images of MPLA-*Eg*Trp-NPs confirmed the nanoparticle size and showed the spherical shape of the particles (Fig 1A). The encapsulated antigen conserved their integrity and their immunogenicity as confirmed by SDS-Page and Western blot analysis on recovered proteins from the pellet of the *Eg*Trp-NPs and *Eg*Trp-NPs-MPLA (Fig 1B and 1C).

**Table 1:** Characterization of nanoparticles for their mean size, size distribution, Zeta potential, and the encapsulation efficiency for loaded proteins and MPLA.

Sample	Particle mean size (nm)	Polydispersity index	Zeta potential (mV)	Antigen Encapsulation efficiency (%)	MPLA Encapsulation efficiency (%)
Blank-NPs	201.5 ± 17.1	0.12 ± 0.03	-3.14 ± 1.40	-	-
*Eg*Trp-NPs	197.7 ± 32.7	0.19 ± 0.11	-5.35 ± 3.31	82.9 ± 4.4	-
*Eg*Trp-NPs-MPLA	185.0 ± 5.8	0.22 ± 0.16	-2.92 ± 1.36	85.6 ± 1.4	85.1 ± 2

The MPLA was introduced in the formulation with four different loading ratio (0.025%, 0.05%, 0.1% and 0.2%) and its encapsulation efficiency was estimated by measuring the MPLA ratio in the pellet and in the supernatant after extraction. The results confirmed that more than 85% MPLA remains in the pellet; showing a very high encapsulation efficiency of MPLA (Table 1).

### Evaluation of nanoparticle internalization by cMoDCs

**Fig 2:**
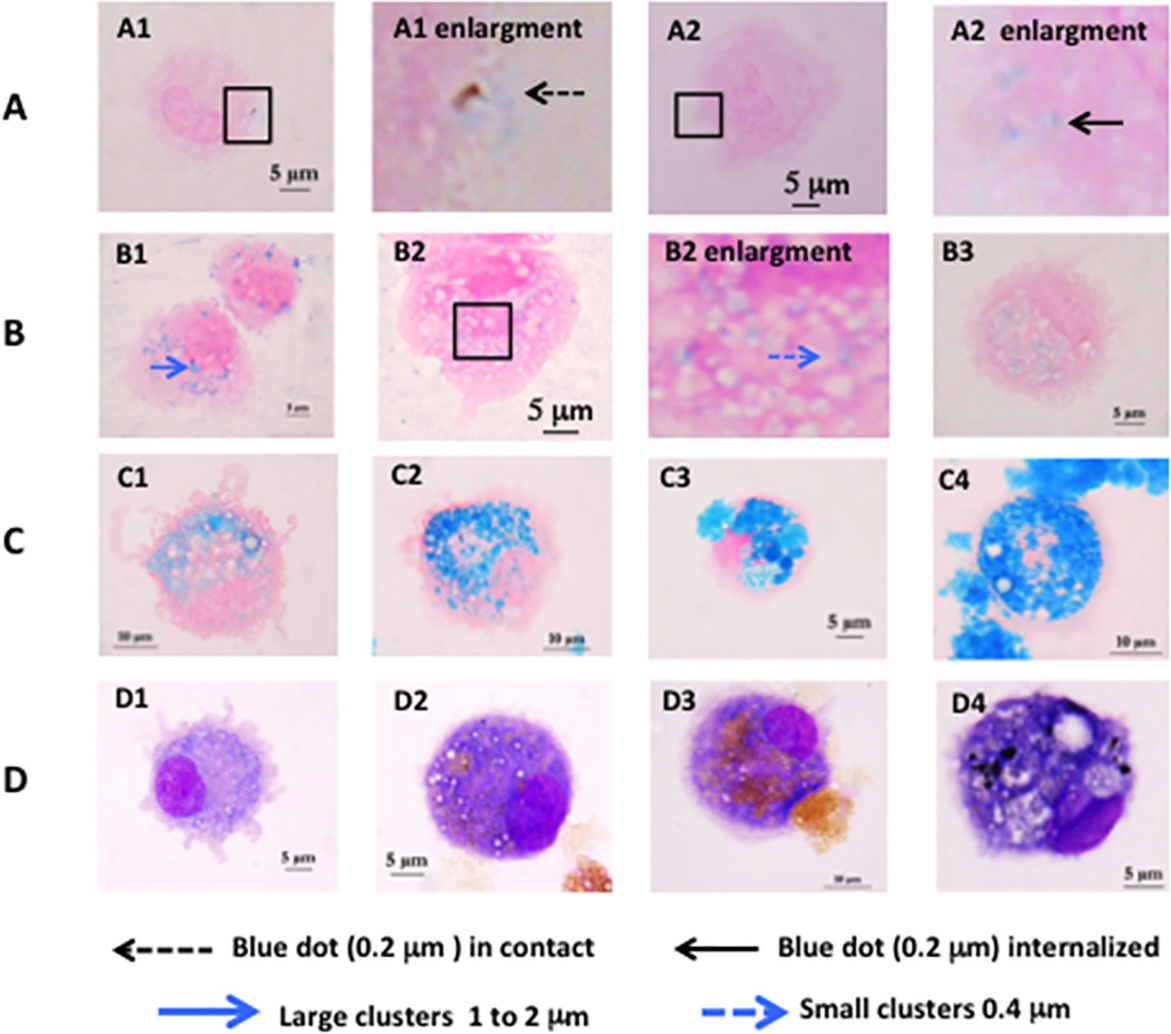
Nanoparticles internalization in cMoDCs observed with Perls staining (A) to (C) and MGG staining (D). (A) after 2h of incubation (A1) with NPs-MPLA 0.1% or (A2) Blank-NPs. (B) after 24h of incubation (B1) with Blank-NPs, (B2) NPs-MPLA 0.1%, (B3) NPs-MPLA 0.025%. (C) after 48h of incubation (C1) with NPs-MPLA 0.025%, (C2) NPs-MPLA 0.05%, (C3) NPs-MPLA 0.1%, (C4) NPs-MPLA 0.2%. (D) after 48 of incubation with (D1) with NPs-MPLA 0.025%, (D2) NPs-MPLA 0.05%, (D3) NPs-MPLA 0.1%, (D4) NPs-MPLA 0.2%.

To evaluate NPs internalization, the nanoparticles were loaded with iron oxide, which can be visualized by Perls staining. After 2 h of incubation with cMoDCs, NPs-MPLA 0.1% were mainly observed at the surface of the dendrites in the form of small blue dots (approximately 0.2 μm diameter) with sizes in accordance with the nanoparticle size (Fig 2A1). Iron still seemed present in the NPs, which were not yet internalized and degraded. For the Blank-NPs condition, some of these small blue dots were observed in the cytoplasm (Fig 2A2). After 24 h of incubation with the Blank-NPs, we observed large blue clusters (approximately 1 to 2 μm diameter) in the cytoplasm of 44% of the cells and small blue clusters (approximately 0.4 μm diameter) in the cytoplasm of 7% of the cells (Fig 2B1). In contrast, with NPs-MPLA, large blue clusters were observed in only 5% of the cells, and small blue clusters were observed in 41% of the cells (Fig 2B2 and 2B3). Clusters (large and small) are larger than the dots observed two hours after contact, suggesting the degradation of the nanoparticles in the cMoDCs and the concentration of iron into endocytic vacuoles. The development of small and large clusters depending on the presence or absence of MPLA suggests that different metabolic pathways are involved.

In order to optimize the MPLA loading ratio, the same experiment was performed with 24 and 48h of contact using four nanoparticle formulations with MPLA at 0.025%, 0.05%, 0.1% and 0.2%. The results showed that the amount of iron accumulation in the cMoDCs was greater at 48 h than at 24 h after contact. With 0.1% and 0.2% MPLA, the cells were completely filled with very large blue clusters, sometimes with extracytoplasmic iron regurgitation. The cells appeared rounded, with no dendrites (Fig 2C3 and 2C4). After MGG staining (Fig 2D3 and 2D4), the cells appeared heavily colored and contained numerous large vacuoles (indication of stress). With 0.05% MPLA (Fig 2C2) the clusters were numerous but did not completely fill the cells and they were not as large as the clusters with the higher MPLA level. The dendrites were small but still observable, and the cells were slightly rounded but did not exhibit a marked stress response (Fig 2D2). With 0.025% MPLA, we observed numerous small- and intermediate-sized blue clusters in the cytoplasm of the cells with long dendrites and normal morphologic aspects (Fig 2C1 and 2D1).

### Cytotoxicity of the nanoparticle formulations

**Fig 3:**
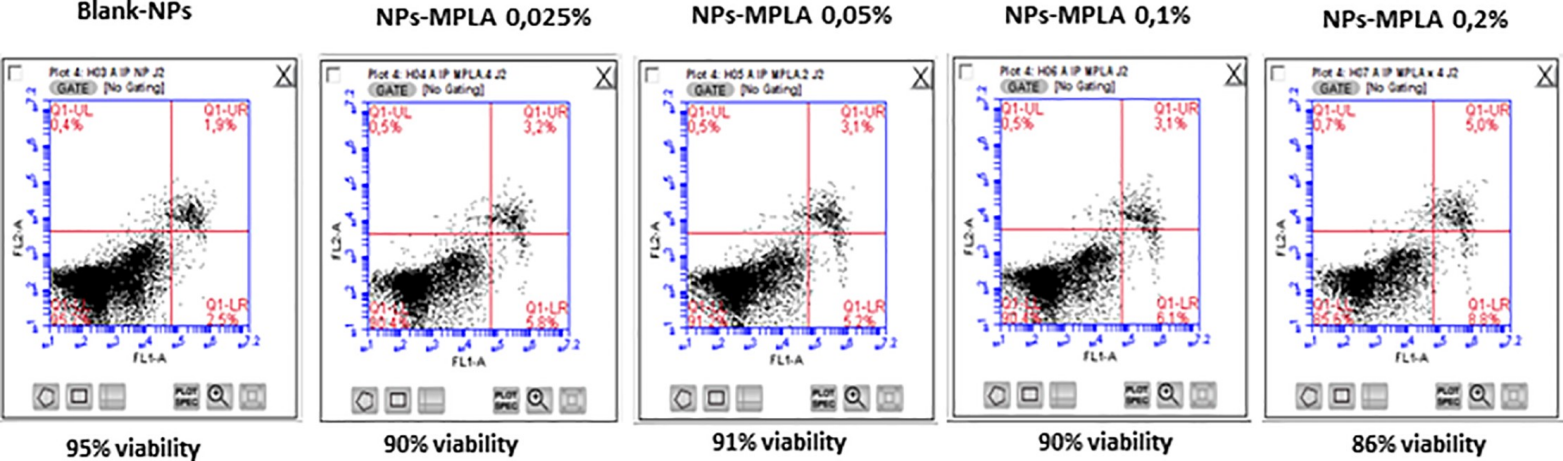
Flow cytometry profiles of cell viability obtained after Annexin V and IP staining of cMoDCs 48 h after incubation with Blank-NPs, NPs-MPLA 0.025%, NPs-MPLA 0.05%, NPs-MPLA 0.1% and NPs-MPLA 0.2%. Annexin V is on FL1-A channel and propidium iodide is on FL2-A channel.

According to our results, the NPs formulations with or without MPLA had no cytotoxic effect on the cMoDCs; cell viability was over 90% in all conditions. Moreover, the MPLA quantities had no effect on cell viability. Although a small, not significant, decrease in cell viability was observed with 0.2% MPLA (Fig 3).

### Evaluation of cMoDC activation markers by flow cytometry

To validate our model, we first evaluated the expression of activation markers on PBMCs (monocyte gate) after Ficoll, on positively selected CD14+ cells before DCs maturation and on cMoDCs at day 6 before challenging. We also evaluated the same activation markers on challenged cMoDCs 24h after incubation with PBS (negative control) or LPS (positive control). In circulating blood and positively selected CD14+ cells, a majority of monocytes naturally expresses CD80, MHCII, and CD40 (S2A–S2C Fig). LPS stimulation only increased the intensity of expression of these markers on the cells surface (increase of MFI 1.2x for CD80, 1.8x for MHC II and 1.7x for CD40 in comparison with PBS challenging). For CD86, a transient expression was observed just after MACS selection then the expression of this marker was not observed on cMoDCs at D6. LPS challenging induced the expression of this marker in 75% of cMoDCs against less than 10% after stimulation with PBS (S2D and S2E Fig). These results strongly suggest that CD86 expression is, in our model, the most reliable marker for cMoDCs activation analysis.

We then evaluated the expression of activation markers on cMoDCs 24 h after incubation with the nanoparticles created with the different MPLA loading ratios, and with positive (LPS) and negative (PBS) controls. As shown in Table 2, the percentages of CD80-, MHC II-, and CD40-positive cells were high and quite similar regardless of the challenge condition. Only a small increase was observed with NPs-MPLA and LPS compared to PBS. In contrast, CD86 expression clearly increased in the cultures treated with LPS, NPs-MPLA 0.025%, or NPs-MPLA 0.05% in comparison with the culture treated with PBS. This increase was far less important for NPs-MPLA 0.1% or 0.2% treatment. Together with the previous results on internalization and cytotoxicity, the nanoparticles created with MPLA at a 0.025% loading ratio seem to be the most appropriate composition for use in vaccine development. This composition was loaded with *Eg*Trp and used in the next part of the study.

**Table 2:** Example of percentages of positive cells for CD80, MHC II, CD40, and CD86, 24 h after contact with PBS (negative control), LPS (positive control at 1μg/ml), and NPs-MPLA at 4 different loading ratios of MPLA.

% of positive cells	PBS	LPS	NPs-MPLA 0.025%	NPs-MPLA 0.05%	NPs-MPLA 0.1%	NPs-MPLA 0.2%
CD80	91.5	95.1	94.5	94.2	95.5	88.8
MHC II	90.5	95.5	94.5	93.9	96.6	94.4
CD40	73.5	81	73.2	73.3	76.6	76.8
CD86	11.2	70.3	64.1	54.6	44.9	43.1

**Fig 4:**
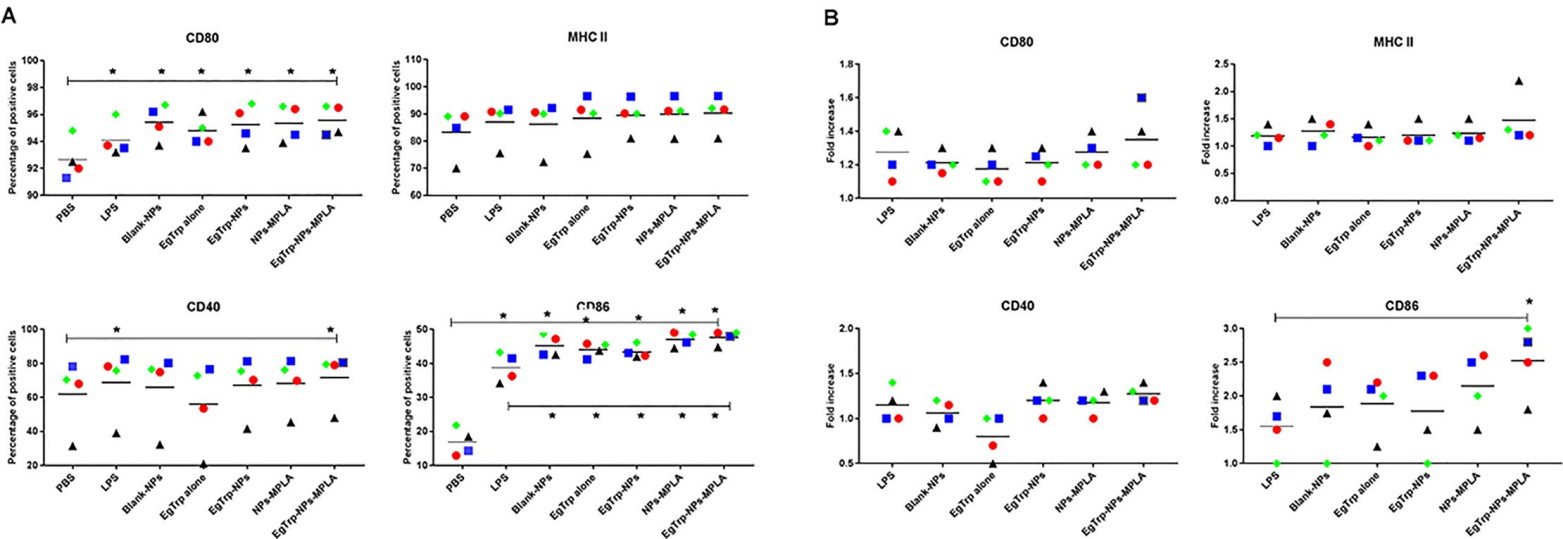
(A) Percentages of positive cells and (B) Fold Increase of MFI compared to PBS for CD80, CD86, MHC II and CD40 expression on the four dogs tested. cMoDCs were challenged with PBS, LPS, Blank-NPs, *Eg*Trp alone, *Eg*Trp-NPs, NPs-MPLA, *Eg*Trp-NPs-MPLA. * Significant difference p ≤ 0.05.

**Fig 5:**
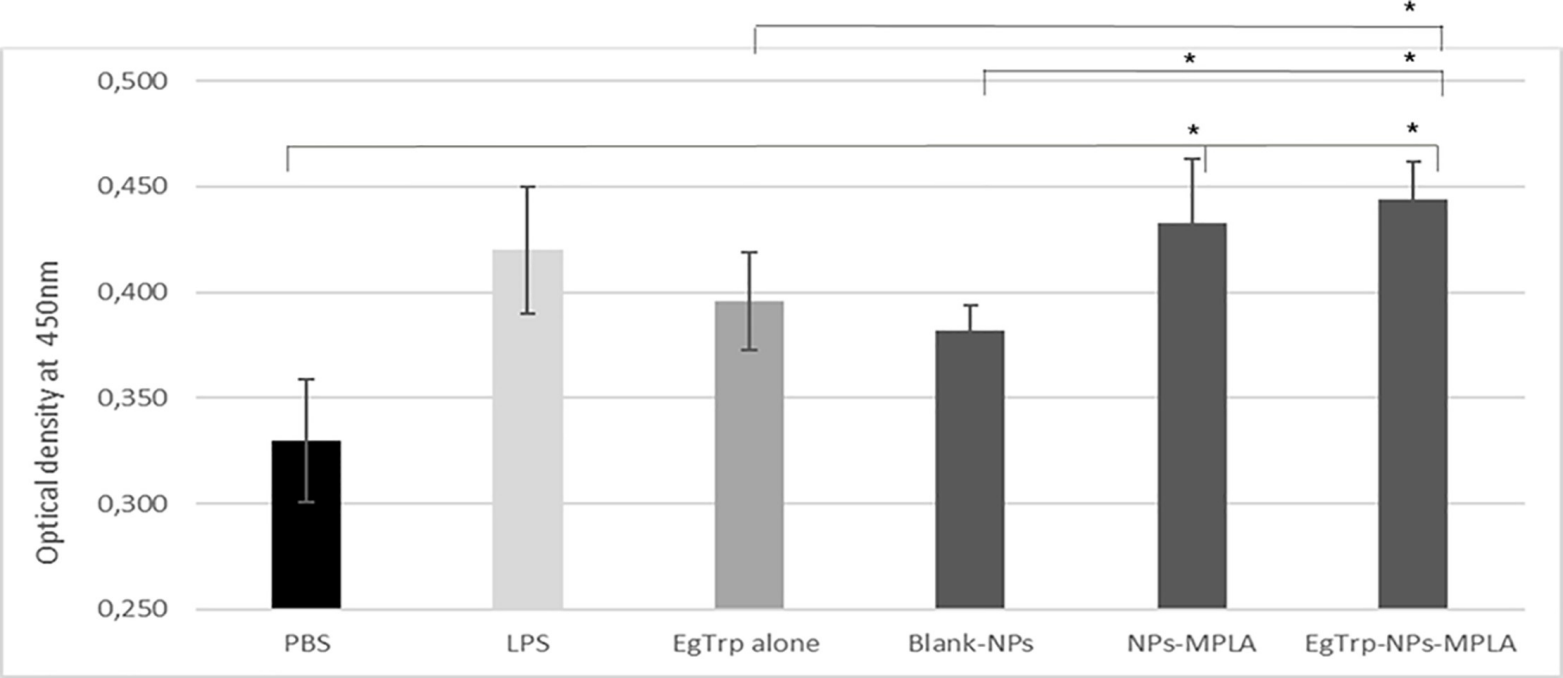
Measure of autologous TCD4+ proliferation by optical density at 450nm after incubation with CCK8. cMoDCs were previously challenged 24 h with PBS (negative control), LPS (positive control), *Eg*Trp alone, Blank-NPs, NPs-MPLA or *Eg*Trp-NPs-MPLA. * Significant difference p ≤ 0.05.

Finally, we evaluated the expression of activation markers on cMoDCs 24 h after challenge with the different formulations. An example of cytometry profiles obtained was shown in supplemental data S3 Fig, and results were analyzed in Fig 4. The percentage of CD80-positive cells was quite similar for all formulations tested (between 94.5% and 97%) and were significantly different from the CD80 expression pattern in the PBS condition (p-value ≤ 0.05). In contrast, the fold increased in the MFI of all the conditions compared to the PBS were similar and ranged between 1.2x and 1.4x. In the cases of MHC II and CD40 expression, three dogs showed very similar results and one dog had percentages of positive cells approximately 20% lower than the others. However, challenges with the different formulations gave similar trends. For MHC II, the percentage of positive cells was approximately 87% for PBS, and increased between 90 and 98% for all the formulations tested. A small increase in the MFI of all the formulations compared to the PBS was also observed (between 1.1 and 1.5). Concerning the percentage of CD40-positive cells, a significant increase was observed for *Eg*Trp-NPs-MPLA in comparison with PBS (p-value ≤ 0.05). In contrast, a clear decrease in the percentage of CD40+ cells was observed with *Eg*Trp alone compared to PBS and all other conditions (Fig 4A). Similar trend was observed with the MFI (Fig 4B). For CD86, significant increases in the percentage of CD86-positive cells were observed after challenging with all formulations (between 34% et 52%) in comparison with challenging with PBS (approximately 15%) (p-value ≤ 0.05). A significant difference was also observed between all formulations with NPs and LPS and between EgTrp-NPs and all formulations with MPLA (p-value ≤ 0.05) (Fig 4A). Concerning the MFI, an increase between 1.4x and 3x was observed for all formulations (Fig 5B). This increase was significant when comparing the complete formulation and LPS (p-value ≤ 0.05).

When the results were compared together, the Blank-NPs and *Eg*Trp-NPs produced similar results whereas the expression of all activation markers increased in the presence of MPLA. Moreover, the complete formulation *Eg*Trp-NPs-MPLA gave the best result compared to all formulations tested.

### Autologous TCD4+ cells proliferation assay

Mixed leukocyte cultures were performed in order to study the ability of cMoDCs challenged with nanoparticles to promote *in vitro* autologous T CD4+ cell priming. Results presented in Fig 5 showed an increase of T cell population in all conditions tested compared to PBS. cMoDCs challenged with LPS and the two formulations with MPLA induced a stronger proliferation than *Eg*Trp alone. Blank-NPs induced a small proliferation. The lymphoproliferative response was significantly higher with cMoDCs challenged with *Eg*Trp-NPs-MPLA than with cMoDCs challenged with PBS, Blank-NPs or *Eg*Trp alone, confirming that the combination of nanoparticles, adjuvant, and antigen improves antigen uptake and presentation to T cells.

## Discussion

In the past decade, the use of nanotechnology in vaccine development has exponentially increased. Indeed, the nanoparticle delivery system enhances antigen processing and improves antigen immunogenicity and stability. The use of adjuvants allows the targeting of specific immune cells, such as dendritic cells, and significantly enhances specific immunological responses [24]. In this system, a good understanding of DCs-NPs interactions is essential for developing efficient nanoparticle vaccines [25]. For example, nanoparticle physical characteristics influence the uptake of NPs by DCs and the processing of antigens through the MHC class I or MHC class II presentation pathways [18]. A number of studies have been carried out *in vitro* to characterize the interactions of different types of NPs with mouse or human dendritic cells [24,25]. In contrast, few studies have been performed on canine cells, and these studies are mainly based on macrophages [26,27]. To our knowledge, this work was the first to study canine dendritic cells and nanoparticle interactions.

For ethical and economic reasons, the use of dogs as animal model most often involves the use of a limited number of animals compared to mouse-type animal model. In our study, 10 dogs had been used to allow all the experiments. However, even if each part of our work could only be done on 3 or 4 dogs, the results obtained were statistically significant.

Because NPs physico-chemical characteristics, such as size, charge, and shape, significantly influences the uptake of NPs by cells, we first studied the internalization efficiency of cMoDCs with our PLA-NPs formulations using iron as a marker. Our results showed that our PLA-NPs were well internalized by canine MoDCs: iron was detected in the cells 2 h after contact, internalization was clearly evident and massive 24 h after contact, and may continue until 48 h after contact. These results are consistent with publications on the internalization characteristics of NPs with a size of approximately 200 nm [28,29]. In canine macrophages, it has been shown that PLGA/OVA-FITC NPs localize within the cytoplasm of the majority of the cells 2 h after contact, and this internalization continues until 24 h [27]. In mouse and human DCs, results have shown that the optimal particle size for fast and efficient uptake is under 500 nm and that internalization occurs in a time- and concentration-dependent manner [28,29].

Moreover, in our work, large or small blue clusters of iron were observed in approximately 50% of the dendritic cells treated with the Blank-NPs and NPs-MPLA after 24 h of contact. This difference in cluster size suggests the involvement of different metabolic pathways: the Blank-NPs are probably internalized through an endocytosis pathway, which leads to the accumulation of iron in the phagosomal compartment, while the NPs-MPLA may be internalized through another specific endocytic-receptor (such as TLR4), which leads to the accumulation of iron in the antigen-processing compartment. In the literature, it has been shown that nanoparticles can be taken up through various pathways depending on their size and the adjuvant used. These different metabolic pathways must influence antigen presentation and need to be considered in the development of a vaccine formulation [30]. For example, Silva et al. (2014) showed that macropinocytosis, clathrin-mediated endocytosis, and caveolin- and lipid raft-dependent endocytosis are involved in the internalization of mannose-grafted PLGA nanoparticles by murine bone marrow-derived DCs. Their formulation allowed the antigen to be presented through both the MHC class I and II pathways, thus inducing a complete immune response [31].

To optimize the MPLA quantity and vaccine production cost, we tested four loading ratios of MPLA (0.025%, 0.05%, 0.1% and 0.2%) in our PLA-NPs formulations. With 0.1% and 0.2% MPLA, morphological observations showed, especially at 48 h, that cMoDCs were completely filled with iron, sometimes with iron extracytoplasmic regurgitation, suggesting a stressed state. However, a cytotoxicity assay showed that none of the PLA-NPs formulations tested had an effect on dendritic cell viability 24 h or 48 h after contact. The absence of nanoparticles- and MPLA-mediated toxicity in cells at the concentrations used (250 μg/mL for NPs and between 6.12 μg/mL and 50 μg/mL for MPLA) was in accordance with the literature. Indeed, studies performed with A549 human lung epithelial cells showed that concentrations of PLA-NPs ranging from 2 to 200 μg/mL or concentrations of PLGA-NPs ranging from 0.01 to 4 mg/mL had no effect on cell viability according to analyses performed with MTT and LDH assays [31,32]. Concerning MPLA, Ismaili et al. found no cytotoxic effects on human monocyte-derived DCs even at 100 μg/mL [19]. Our morphological observations are most likely linked to iron toxicity, as iron accumulated to high levels in the cytoplasm of cells with high MPLA concentrations, generating a rejection defense mechanism. In conclusion, because our nanoparticles containing 0.025% MPLA were well internalized, had no impact on cell viability and further limited the vaccine production cost, this concentration was selected for the second part of the study.

The internalization of nanoparticles into immature MoDCs could be considered a “danger signal” leading to the maturation of the dendritic cells and expression of activation markers as CD80, MHCII, CD40, and CD86. We observed that even before the challenge, our cMoDCS strongly expressed three of these markers: CD80, MHC II, and CD40. This is in accordance with dog literature data [33–35] and suggest that cMoDCs generated *in vitro* are already partially mature and activated even if they maintain their ability to strongly internalize antigen. Stimulation of our cells with LPS induces increased expression of these markers and, more interestingly, elicits CD86 expression. These results are consistent with those published about humans, mouse, and canine dendritic cells [23], and validates the particular interest in the dog model of CD86 as a DCs activation marker.

We then analyzed the expression of CD40, CD80, CD86, and MHC II, after 24 h of incubation with one of the following formulations: *EgTrp* alone, Blank-NPs, NPs-MPLA, *Eg*Trp-NPs, and *Eg*Trp-NPs-MPLA. Our results showed that both the percentage of CD80, MHC II, and CD40 positives cells and the fold increase of MFI were slightly higher in the *Eg*Trp-NPs-MPLA condition than in the LPS condition. More important, the percentage of CD86 positives cells and the fold increase of MFI were significantly higher in the *Eg*Trp-NPs-MPLA condition than in the LPS condition (Fig 4A and 4B). Results obtained with the *Eg*Trp-NPs-MPLA formulation were also better, or significantly better, than those obtained with all others formulations.

Our results also showed that Blank-NPs were able to enhance CD86 expression on cMoDCs. In publications, contradictory results are observed. Uto et al. showed that PGA nanoparticles induced a dose-dependent increased expression of CD40, CD86, and MHC class I on mouse DCs [36]. In contrast, Margaroni et al. describe that empty PLGA NPs were unable to induce mouse DCs maturation [37]. Thus, DCs activation seems to be dependent of the nanoparticles used. In the same way, Margaroni et al. observed a better activation of DCs at 24h with the formulation Antigen-NPs than with Blank-NPs in their mouse model [37]. In our canine model, Blank-NPs and EgTrp-NPs gave similar results at 24h. We hypothesized that at this time, in our model, PLA-NPs have been internalized but the antigen has not yet released from the nanoparticle.

In the case of *Eg*Trp alone, for which the antigen is in a soluble form, we observed a very moderate activation of dendritic cells compared to all other formulations tested. In fact, DCs internalize soluble antigens by pinocytosis whereas particulate antigens (as antigen enclosed into NPs) were internalized by endocytosis, a pathway considered more efficient in induction of immune responses. Indeed, antigens uptake is described to increase up to 30-fold for particulate antigens in comparison to soluble antigens [15]. This difference in cellular pathways may explain our results.

In the case of formulations with MPLA, our results show a better response with NPs-MPLA than with Blank-NPs, in accordance with previous publications [37,38]. For example, Margaroni et al. showed that their vaccine formulation with PLGA nanoparticles and MPLA induced significantly higher expression of CD40, CD80, CD86, and MHC class II on mouse DCs, and a more efficient IL-12 and IL-10 production than NPs without MPLA [37]. The importance of MPLA in vaccine formulations has been confirmed *in vivo* in a mouse model [39,40]. Sarti et al. showed that their PLGA-MPLA-OVA vaccine formulation induced a stronger IgG immune response than OVA in PBS solution alone, or PLGA-OVA without MPLA [40].

To complete this work we analyzed the ability of these activated cMoDCs to promote *in vitro* autologous T CD4+ cell priming. Our results showed that the lymphoproliferative response was significantly higher when cells were challenged with *Eg*Trp-NPs-MPLA than with PBS, Blank-NPs, or *Eg*Trp alone (Fig 5). In these experiments, we also observed that the Blank-NPs were able to induce a small proliferation of T cells *in vitro*. In publications, contradictory results are observed. Uto et al. observed that Poly (γ-Glutamic Acid) Nanoparticles treated DCs induced allogeneic T cells proliferation in a dose dependent manner in mouse model [36]. In contrast, Margaroni et al. reported that mouse DCs stimulated with empty PLGA NPs were unable to induce T cell proliferation [37]. Thus, as the activation of DCs, T cells proliferation seem to be dependent on the nanoparticles used.

In conclusion, these results confirmed *in vitro* that the combination of PLA-NPs as vehicle, MPLA as adjuvant and the recombinant *Eg*Trp protein as immunogen induce maturation of cMoDC and autologous T CD4+ proliferation more efficiently than the immunogen alone. These results are promising for the development of a vaccine against *Echinococcus granulosus*. The development of the complete vaccine formulation is now necessary before performing tests in dog model *in vivo*.

## Supporting information

S1 Fig cMoDCs characterization and comparison of the two protocols used: Adherent protocol versus positive selection of CD14+ cells with MACS system. (A) Morphological changes observed between adherent cells at D1, CD14 positive cells at D0, and cMoDCs at D6 observed by light microscopy after cytocentrifugation and May Grunewald Giemsa staining. (B) Flow cytometry profiles obtained on PBMC at D0 (gate monocyte), CD14+ selected cells at D0, and cMoDCs at D6. **A**: Morphological changes observed after MGG staining of the adherent cells or the CD14+ PBMCs at day 1 and the cMoDCs at day 6 are similar. At day 1, cells were small and round, with ovoid or kidney-shaped nuclei and moderately expanded cytoplasm. At D6, cells were larger and displayed a more expanded cytoplasm with thin cytoplasmic projections (dendrites) radiating from the surface. **B:** In the flow cytometry profiles, PBMC cells in the monocyte gate at day 0 appear to be of small size (FSC) with low granulometry (SSC), whereas at day 6, we observed strong increases in both parameters, confirming the morphological changes. The adherence method allowed to obtain 1 x 10^6^ cMoDCs at day 6 with a significant number of other cell types, such as lymphocytes, polymorphonuclear cells and platelets. Positive magnetic-sorting selection method allowed to obtain 5 x 10^5^ CD14+ cells but unlike the adherence protocol, the purity of the cMoDCs obtained on day 6 was higher than 90%.
(PDF)

S2 Fig Cytometry profiles of CD14, CD80, CD86, MHC II and CD40 expression obtained on (A) canine monocytes in PBMC, (B) positively selected CD14+ at D0, (C) cMoDCs at D6 before challenging, (D) cMoDCs 24h after challenging with PBS (negative control) or (E) LPS (positive control). **A:** Among the PBMCs, a majority of the cells in the monocyte gate (60–70%) spontaneously expressed CD14, CD80, MHC II and CD40 at a high level but they did not express CD86. **B:** After MACS selection, more than 90% of the cells were CD14+ and expressed CD80, MHC II and CD40 at a high level. Surprisingly approximately 30% of these cells also expressed CD86, suggesting that the positive selection process with the anti-CD14 antibody induced a cellular activation. **C:** After cMoDCs differentiation, the expression of CD14, CD80, MHC II and CD40 remained positive and high for a large majority of the cells (above 90%), while CD86 expression decreased under 10%. **D and E:** After 24 h of incubation with LPS, approximately 75% of the cMoDCs expressed CD86 compared to less than 10% after the incubation with PBS. The expression of CD80, MHC II and CD40 was quite similar according to the percentage of positive cells in the culture stimulated with LPS compared with PBS; however, an increase in MFI was detected for all of these markers in cells stimulated with LPS.
(PDF)

S3 Fig Example of cytometry profiles of CD80, MHC II, CD40 and CD86 expression obtained on cMoDCs 24h after of challenge with (A) PBS, (B) LPS, (C) Blank-NPs, (D) *Eg*Trp alone, (E) *Eg*Trp-NPs, (F) NPs-MPLA and (G) *Eg*Trp-NPs-MPLA.
(PDF)

## References

[1] Moro P, Schantz PM. Echinococcosis: a review. Int J Infect Dis. 2009 3;13(2):125–33. 10.1016/j.ijid.2008.03.037 18938096

[2] Pourseif MM, Moghaddam G, Saeedi N, Barzegari A, Dehghani J, Omidi Y. Current status and future prospective of vaccine development against Echinococcuc granulosus. Biologicals. 2018 1;51:1–11. 10.1016/j.biologicals.2017.10.003 29100669

[3] Craig PS, Hegglin D, Lightowlers MW, Torgerson PR, Wang Q. Echinococcosis: Control and Prevention. Adv Parasitol. 2017;96:55–158. 10.1016/bs.apar.2016.09.002 28212791

[4] Lightowlers MW, Lawrence SB, Gauci CG, Young J, Ralston MJ, Maas D, et al Vaccination against hydatidosis using a defined recombinant antigen. Parasite Immunol. 1996 9;18(9):457–62. 10.1111/j.1365-3024.1996.tb01029.x 9226681

[5] Heath DD, Robinson C, Shakes T, Huang Y, Gulnur T, Shi B, et al Vaccination of bovines against Echinococcus granulosus (cystic echinococcosis). Vaccine. 2012 4 26;30(20):3076–81. 10.1016/j.vaccine.2012.02.073 22406459

[6] Pan W, Chen D-S, Lu Y-J, Xu H-W, Hao W-T, Zhang Y-W, et al Genetic diversity and phylogenetic analysis of EG95 sequences of Echinococcus granulosus: Implications for EG95 vaccine application. Asian Pac J Trop Med. 2017 5;10(5):524–7. 10.1016/j.apjtm.2017.05.011 28647192

[7] Zhang W, Zhang Z, Shi B, Li J, You H, Tulson G, et al Vaccination of dogs against Echinococcus granulosus, the cause of cystic hydatid disease in humans. J Infect Dis. 2006 10 1;194(7):966–74. 10.1086/506622 16960785

[8] Zhang Z, Guo G, Li J, Shi BX, Zhao L, Guo BP, et al Dog vaccination with EgM proteins against Echinococcus granulosus. Infect Dis Poverty. 2018; 7: 61 10.1186/s40249-018-0425-4 29895318 PMC5998577

[9] Fraize M, Sarciron ME, Azzouz S, Issaadi N, Bosquet G, Petavy AF. Immunogenicity of two Echinococcus granulosus antigens EgA31 and EgTrp in mice. Parasitol Res. 2005 5;96(2):113–20. 10.1007/s00436-005-1322-x 15824902

[10] Petavy A-F, Hormaeche C, Lahmar S, Ouhelli H, Chabalgoity A, Marchal T, et al An oral recombinant vaccine in dogs against Echinococcus granulosus, the causative agent of human hydatid disease: a pilot study. PLoS Negl Trop Dis. 2008 1 16;2(1):e125 10.1371/journal.pntd.0000125 18235847 PMC2217674

[11] Calderon-Gonzalez R, Marradi M, Garcia I, Petrovsky N, Alvarez-Dominguez C. Novel nanoparticle vaccines for Listeriosis. Hum Vaccines Immunother. 2015;11(10):2501–3. 10.1080/21645515.2015.1063756 PMC4635887 26252360

[12] Moon JJ, Suh H, Polhemus ME, Ockenhouse CF, Yadava A, Irvine DJ. Antigen-displaying lipid-enveloped PLGA nanoparticles as delivery agents for a Plasmodium vivax malaria vaccine. PloS One. 2012;7(2):e31472 10.1371/journal.pone.0031472 22328935 PMC3273465

[13] Hunsawong T, Sunintaboon P, Warit S, Thaisomboonsuk B, Jarman RG, Yoon I-K, et al A novel dengue virus serotype-2 nanovaccine induces robust humoral and cell-mediated immunity in mice. Vaccine. 2015 3 30;33(14):1702–10. 10.1016/j.vaccine.2015.02.016 25701315

[14] Tyler B, Gullotti D, Mangraviti A, Utsuki T, Brem H. Polylactic acid (PLA) controlled delivery carriers for biomedical applications. Adv Drug Deliv Rev. 2016 12 15;107:163–75. 10.1016/j.addr.2016.06.018 27426411

[15] Gregory AE, Titball R, Williamson D. Vaccine delivery using nanoparticles. Front Cell Infect Microbiol. 2013;3:13 10.3389/fcimb.2013.00013 23532930 PMC3607064

[16] Smith DM, Simon JK, Baker JRJ. Applications of nanotechnology for immunology. Nat Rev Immunol. 2013 8;13(8):592–605. 10.1038/nri3488 23883969 PMC7097370

[17] Sehgal K, Dhodapkar KM, Dhodapkar MV. Targeting human dendritic cells in situ to improve vaccines. Immunol Lett. 2014 11;162(1 Pt A):59–67. 10.1016/j.imlet.2014.07.004 25072116 PMC4506641

[18] Reddy ST, Swartz MA, Hubbell JA. Targeting dendritic cells with biomaterials: developing the next generation of vaccines. Trends Immunol. 2006 12;27(12):573–9. 10.1016/j.it.2006.10.005 17049307

[19] Ismaili J, Rennesson J, Aksoy E, Vekemans J, Vincart B, Amraoui Z, et al Monophosphoryl lipid A activates both human dendritic cells and T cells. J Immunol Baltim Md 1950. 2002 1 15;168(2):926–32. 10.4049/jimmunol.168.2.926 11777991

[20] Esteves A, Senorale M, Ehrlich R. A tropomyosin gene is differentially expressed in the larval stage of Echinococcus granulosus. Parasitol Res. 2003 4;89(6):501–2. 10.1007/s00436-002-0791-4 12658463

[21] Bilati U, Allémann E, Doelker E. Poly(D,L-lactide-co-glycolide) protein-loaded nanoparticles prepared by the double emulsion method—processing and formulation issues for enhanced entrapment efficiency. J Microencapsul. 2005 3;22(2):205–14. 10.1080/02652040400026442 16019905

[22] Bonnefont-Rebeix C, de Carvalho CM, Bernaud J, Chabanne L, Marchal T, Rigal D. CD86 molecule is a specific marker for canine monocyte-derived dendritic cells. Vet Immunol Immunopathol. 2006 1 15;109(1–2):167–76. 10.1016/j.vetimm.2005.08.027 16202456

[23] Qeska V, Baumgartner W, Beineke A. Species-specific properties and translational aspects of canine dendritic cells. Vet Immunol Immunopathol. 2013 2 15;151(3–4):181–92. 10.1016/j.vetimm.2012.12.003 23280245

[24] Klippstein R, Pozo D. Nanotechnology-based manipulation of dendritic cells for enhanced immunotherapy strategies. Nanomedicine Nanotechnol Biol Med. 2010 8;6(4):523–9. 10.1016/j.nano.2010.01.001 20085824

[25] Zhao L, Seth A, Wibowo N, Zhao C-X, Mitter N, Yu C, et al Nanoparticle vaccines. Vaccine. 2014 1 9;32(3):327–37. 10.1016/j.vaccine.2013.11.069 24295808

[26] Derman S, Mustafaeva ZA, Abamor ES, Bagirova M, Allahverdiyev A. Preparation, characterization and immunological evaluation: canine parvovirus synthetic peptide loaded PLGA nanoparticles. J Biomed Sci. 2015 10 20;22:89 10.1186/s12929-015-0195-2 26482775 PMC4617543

[27] Guldner D, Hwang JK, Cardieri MCD, Eren M, Ziaei P, Norton MG, et al In Vitro Evaluation of the Biological Responses of Canine Macrophages Challenged with PLGA Nanoparticles Containing Monophosphoryl Lipid A. PloS One. 2016;11(11):e0165477 10.1371/journal.pone.0165477 27835636 PMC5105989

[28] Foged C, Brodin B, Frokjaer S, Sundblad A. Particle size and surface charge affect particle uptake by human dendritic cells in an in vitro model. Int J Pharm. 2005 7 25;298(2):315–22. 10.1016/j.ijpharm.2005.03.035 15961266

[29] Xiang SD, Scholzen A, Minigo G, David C, Apostolopoulos V, Mottram PL, et al Pathogen recognition and development of particulate vaccines: does size matter? Methods San Diego Calif. 2006 9;40(1):1–9 10.1016/j.ymeth.2006.05.016 16997708

[30] Oh N, Park J-H. Endocytosis and exocytosis of nanoparticles in mammalian cells. Int J Nanomedicine. 2014;9 Suppl 1:51–63. 24872703 10.2147/IJN.S26592 PMC4024976

[31] Silva JM, Vandermeulen G, Oliveira VG, Pinto SN, Rodrigues C, Salgado A, et al Development of functionalized nanoparticles for vaccine delivery to dendritic cells: a mechanistic approach. Nanomed. 2014 12;9(17):2639–56. 10.2217/nnm.14.135 25529568

[32] Grabowski N, Hillaireau H, Vergnaud J, Santiago LA, Kerdine-Romer S, Pallardy M, et al Toxicity of surface-modified PLGA nanoparticles toward lung alveolar epithelial cells. Int J Pharm. 2013 10 1;454(2):686–94. 10.1016/j.ijpharm.2013.05.025 23747506

[33] Wang Y-S, Chi K-H, Liao K-W, Liu C-C, Cheng C-L, Lin Y-C, et al Characterization of canine monocyte-derived dendritic cells with phenotypic and functional differentiation. Can J Vet Res. 2007 7;71(3):165–74. 17695590 PMC1899861

[34] Bund D, Buhmann R, Gokmen F, Kremser A, Dreyssig J, Kolb HJ, et al Canine-DCs using different serum-free methods as an approach to provide an animal-model for immunotherapeutic strategies. Cell Immunol. 2010;263(1):88–98. 10.1016/j.cellimm.2010.03.003 20347071

[35] Ibisch C, Pradal G, Bach JM, Lieubeau B. Functional canine dendritic cells can be generated in vitro from peripheral blood mononuclear cells and contain a cytoplasmic ultrastructural marker. J Immunol Methods. 2005 3;298(1–2):175–82. 10.1016/j.jim.2005.02.001 15847807

[36] Uto T, Wang X, Sato K, Haraguchi M, Akagi T, Akashi M, et al Targeting of antigen to dendritic cells with poly (gamma-glutamic acid) nanoparticles induces antigen-specific humoral and cellular immunity. J Immunol. 2007 3 1;178(5):2979–86. 10.4049/jimmunol.178.5.2979 17312143

[37] Margaroni M, Agallou M, Kontonikola K, Karidi K, Kammona O, Kiparissides C, et al PLGA nanoparticles modified with a TNFalpha mimicking peptide, soluble Leishmania antigens and MPLA induce T cell priming in vitro via dendritic cell functional differentiation. Eur J Pharm Biopharm. 2016 8;105:18–31. 10.1016/j.ejpb.2016.05.018 27235727

[38] Elamanchili P, Diwan M, Cao M, Samuel J. Characterization of poly (D,L-lactic-co-glycolic acid) based nanoparticulate system for enhanced delivery of antigens to dendritic cells. Vaccine. 2004 6 23;22(19):2406–12. 10.1016/j.vaccine.2003.12.032 15193402

[39] Weilhammer DR, Blanchette CD, Fischer NO, Alam S, Loots GG, Corzett M, et al The use of nanolipoprotein particles to enhance the immunostimulatory properties of innate immune agonists against lethal influenza challenge. Biomaterials. 2013 12;34(38):10305–18. 10.1016/j.biomaterials.2013.09.038 24075406 PMC7172747

[40] Sarti F, Perera G, Hintzen F, Kotti K, Karageorgiou V, Kammona O, et al In vivo evidence of oral vaccination with PLGA nanoparticles containing the immunostimulant monophosphoryl lipid A. Biomaterials. 2011 6;32(16):4052–7. 10.1016/j.biomaterials.2011.02.011 21377204

