# Supplementary material for: In vitro evaluations on canine monocyte-derived dendritic cells of a nanoparticles delivery system for vaccine antigen against Echinococcus granulosus
Source: PLoS One. 2020 Feb 26;15(2):e0229121. doi: 10.1371/journal.pone.0229121 (PMC7043750; doi:10.1371/journal.pone.0229121)
Supplement: S1 Fig — (A) Morphological changes observed between adherent cells at D1, CD14 positive cells at D0, and cMoDCs at D6 observed by light microscopy after cytocentrifugation and May Grunewald Giemsa staining. (B) Flow cytometry profiles obtained on PBMC at D0 (gate monocyte), CD14+ selected cells at D0, and cMoDCs at D6. A: Morphological changes observed after MGG staining of the adherent cells or the CD14+ PBMCs at day 1 and the cMoDCs at day 6 are similar. At day 1, cells were small and round, with ovoid or kidney-shaped nuclei and moderately expanded cytoplasm. At D6, cells were larger and displayed a more expanded cytoplasm with thin cytoplasmic projections (dendrites) radiating from the surface. B: In the flow cytometry profiles, PBMC cells in the monocyte gate at day 0 appear to be of small size (FSC) with low granulometry (SSC), whereas at day 6, we observed strong increases in both parameters, confirming the morphological changes. The adherence method allowed to obtain 1 x 10^6^ cMoDCs at day 6 with a significant number of other cell types, such as lymphocytes, polymorphonuclear cells and platelets. Positive magnetic-sorting selection method allowed to obtain 5 x 10^5^ CD14+ cells but unlike the adherence protocol, the purity of the cMoDCs obtained on day 6 was higher than 90%. (PDF) [file pone.0229121.s001.pdf]

Figure S1: cMoDCs characterization and comparison of the two protocols used: adherent protocol versus positive selection of CD14+ cells with MACS system**.**

(A) Morphological changes observed between adherent cells at D1 or CD14 positive cells at D0 and cMoDCs at D6 observed by light microscopy after cytocentrifugation and May Grunewald Giemsa staining.
(B) Flow cytometry profiles obtained on PBMC at D0 (gate monocyte), CD14+ selected cells at D0 and cMoDCs at D6.

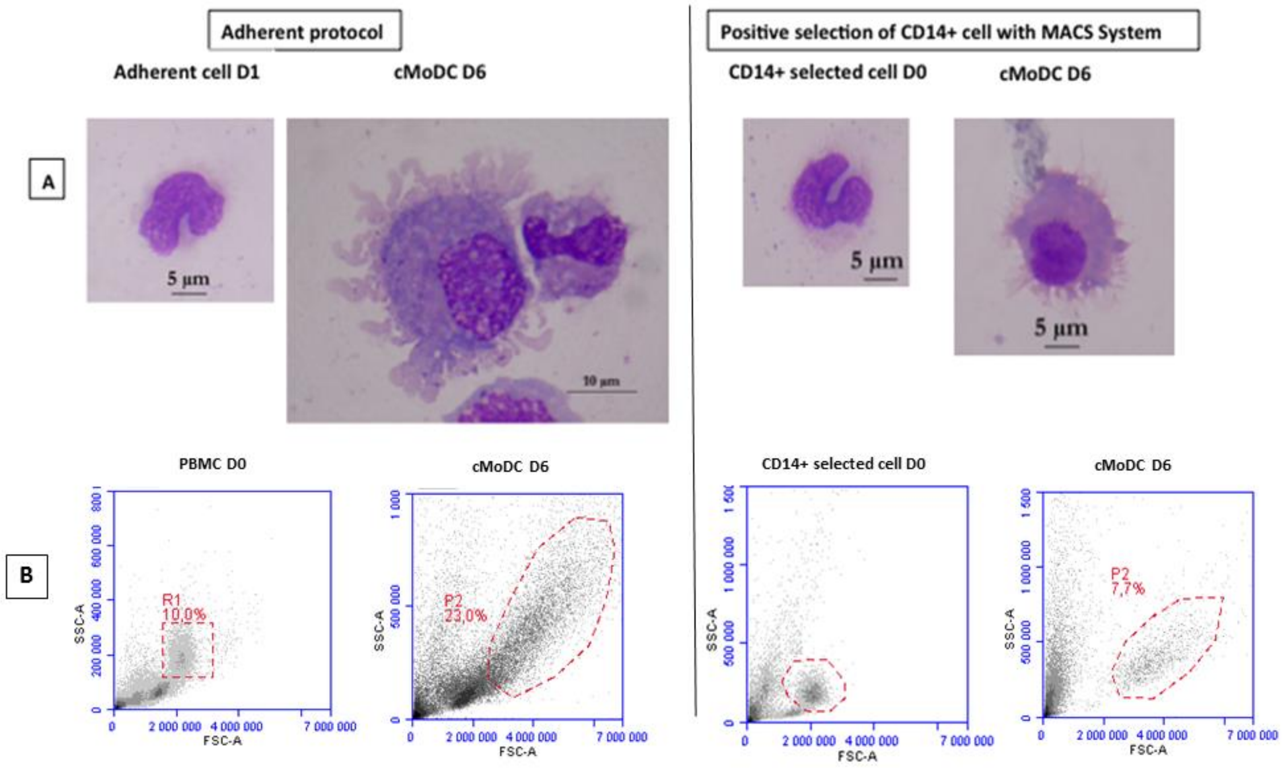

Fig S1A: Morphological changes observed that after MGG staining of the adherent cells, the CD14+ PBMCs at day 1 and the cMoDCs at day 6 are similar. At day 1, cells were small, round, with ovoid or kidney-shaped nuclei, and moderately expanded cytoplasm. At D6, cells were larger and displayed a more expanded cytoplasm with thin cytoplasmic projections (dendrites) radiating from the surface.

Fig S1B: In the flow cytometry profiles, PBMC cells in the monocyte gate at day 0 appear to be of small size (FSC) with low granulometry (SSC), whereas at day 6, we observed strong increases in both parameters, confirming the morphological changes. The adherence method allowed to obtain 1 x 10^6 cMoDCs at day 6 with a significant number of other cell types, such as lymphocytes, polymorphonuclear cells and platelets. Positive magnetic-sorting selection method allowed to obtain 5 x 10^5 CD14+ cells but unlike the adherence protocol, the purity of the cMoDCs obtained on day 6 was higher than 90%.
