# Supplementary material for: In vitro evaluations on canine monocyte-derived dendritic cells of a nanoparticles delivery system for vaccine antigen against Echinococcus granulosus
Source: PLoS One. 2020 Feb 26;15(2):e0229121. doi: 10.1371/journal.pone.0229121 (PMC7043750; doi:10.1371/journal.pone.0229121)
Supplement: S2 Fig — Cytometry profiles of CD14, CD80, CD86, MHC II and CD40 expression obtained on (A) canine monocytes in PBMC, (B) positively selected CD14+ at D0, (C) cMoDCs at D6 before challenging, (D) cMoDCs 24h after challenging with PBS (negative control) or (E) LPS (positive control). A: Among the PBMCs, a majority of the cells in the monocyte gate (60–70%) spontaneously expressed CD14, CD80, MHC II and CD40 at a high level but they did not express CD86. B: After MACS selection, more than 90% of the cells were CD14+ and expressed CD80, MHC II and CD40 at a high level. Surprisingly approximately 30% of these cells also expressed CD86, suggesting that the positive selection process with the anti-CD14 antibody induced a cellular activation. C: After cMoDCs differentiation, the expression of CD14, CD80, MHC II and CD40 remained positive and high for a large majority of the cells (above 90%), while CD86 expression decreased under 10%. D and E: After 24 h of incubation with LPS, approximately 75% of the cMoDCs expressed CD86 compared to less than 10% after the incubation with PBS. The expression of CD80, MHC II and CD40 was quite similar according to the percentage of positive cells in the culture stimulated with LPS compared with PBS; however, an increase in MFI was detected for all of these markers in cells stimulated with LPS. (PDF) [file pone.0229121.s002.pdf]

Figure S2: Cytometry profiles of CD14, CD80, CD86, MHC II and CD40 expression

Obtained on (A) canine monocytes in PBMC, (B) positively selected CD14+ at D0, (C) cMoDCs at D6 before challenging, (D) cMoDCs 24h after challenging with PBS (negative control) or (E) LPS (positive control).

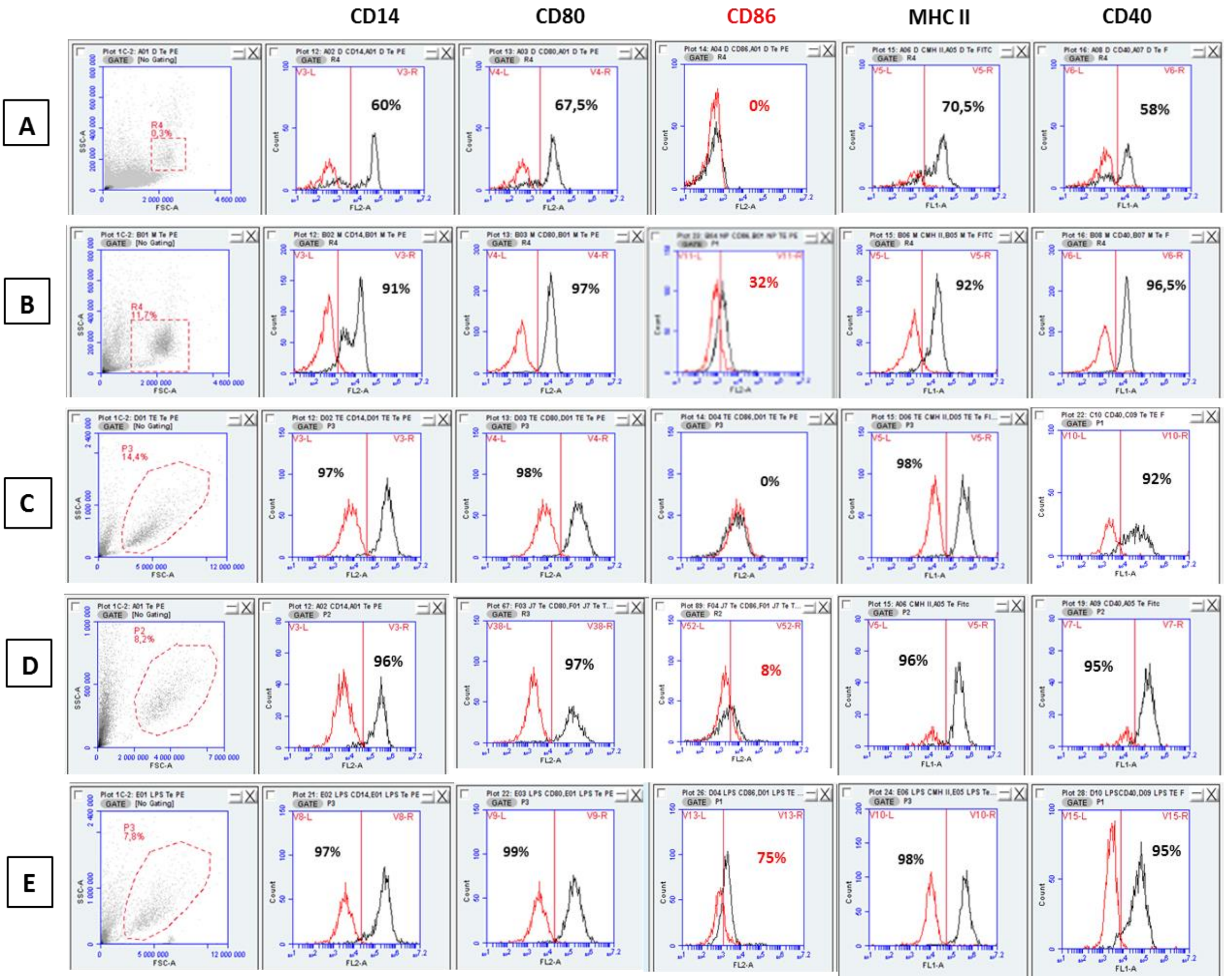

Fig S2A: Among the PBMCs, a majority of the cells in the monocyte gate (60-70%) spontaneously expressed CD14, CD80, MHC II and CD40 at a high level but they did not express CD86

Fig S2B: After MACS selection, more than 90% of the cells were CD14+ and expressed CD80, MHC II and CD40 at a high level. Surprisingly approximately 30% of these cells also expressed CD86, suggesting that the positive selection process with the anti-CD14 antibody induced a cellular activation.

Fig 2C: After cMoDCs differentiation, the expression of CD14, CD80, MHC II and CD40 remained positive and high for a large majority of the cells (above 90%), while CD86 expression decreased under 10%.

Fig S2D and S2E: After 24 h of incubation with LPS, approximately 75% of the cMoDCs expressed CD86 compared to less than 10% after the incubation with PBS. The expression of CD80, MHC II and CD40 was quite similar according to the percentage of positive cells in the culture stimulated with LPS compared with PBS; however, an increase in MFI was detected for all of these markers in cells stimulated with LPS.
