# Supplementary material for: In vitro evaluations on canine monocyte-derived dendritic cells of a nanoparticles delivery system for vaccine antigen against Echinococcus granulosus
Source: PLoS One. 2020 Feb 26;15(2):e0229121. doi: 10.1371/journal.pone.0229121 (PMC7043750; doi:10.1371/journal.pone.0229121)

Figure S3: Example of cytometry profiles of CD80, MHC II, CD40 and CD86 expression obtained on cMoDCs 24h after of challenge with (A) PBS, (B) LPS, (C) Blank-NPs, (D) *Eg*Trp alone, (E) *Eg*Trp-NPs, (F) NPs-MPLA and (G) *Eg*Trp-NPs-MPLA.

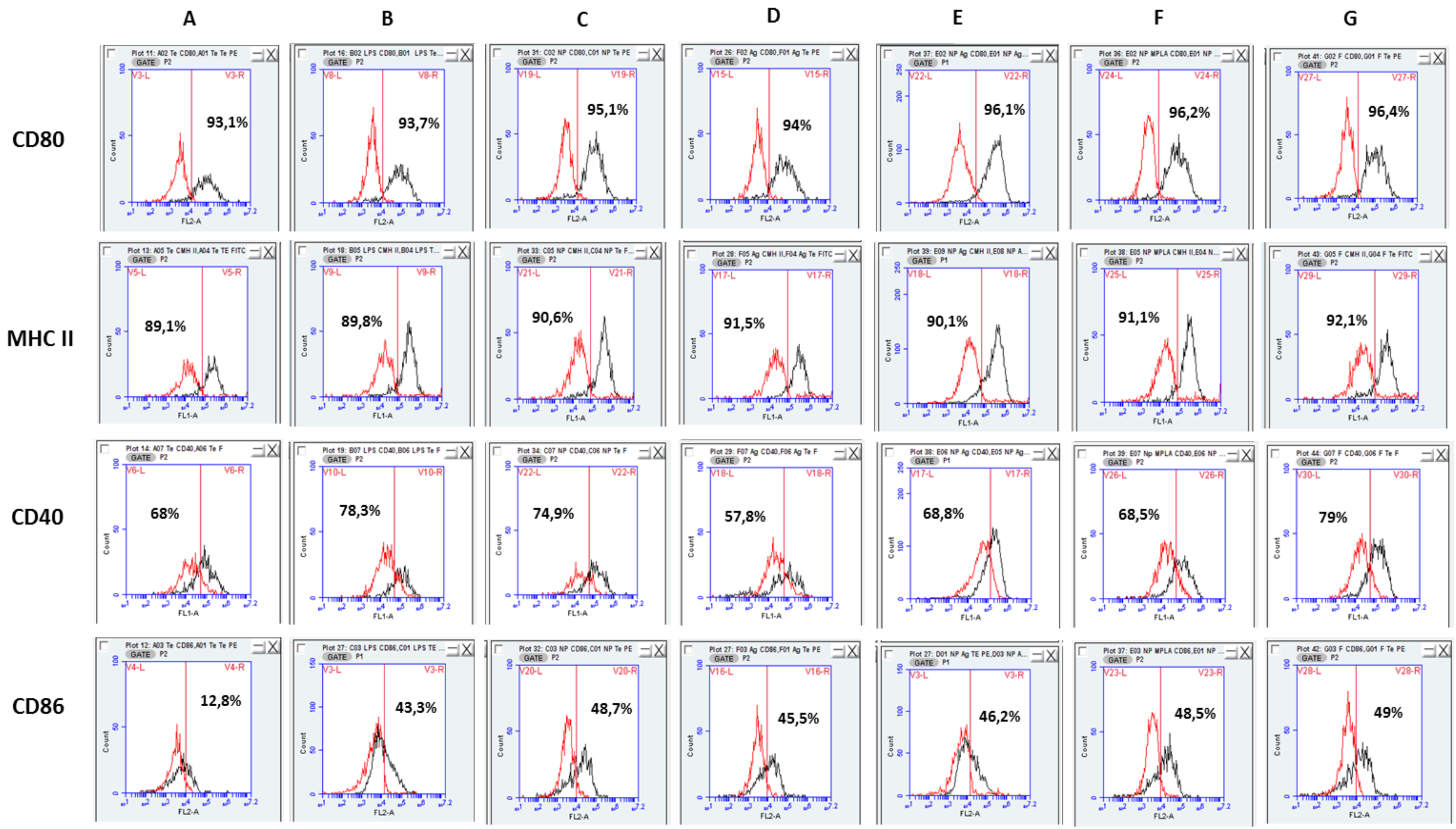

Supplement: S3 Fig — Example of cytometry profiles of CD80, MHC II, CD40 and CD86 expression obtained on cMoDCs 24h after of challenge with (A) PBS, (B) LPS, (C) Blank-NPs, (D) EgTrp alone, (E) EgTrp-NPs, (F) NPs-MPLA and (G) EgTrp-NPs-MPLA. (PDF) [file pone.0229121.s003.pdf]
